# Evolutionary cores of domain co-occurrence networks

**DOI:** 10.1186/1471-2148-5-24

**Published:** 2005-03-23

**Authors:** Stefan Wuchty, Eivind Almaas

**Affiliations:** 1 Northwestern Institute of Complexity, Northwestern University, Evanston, IL, USA; 2 Center for Complex Network Research and Department of Physics, University of Notre Dame, Notre Dame, IN 46556, USA

## Abstract

**Background:**

The modeling of complex systems, as disparate as the World Wide Web and the cellular metabolism, as networks has recently uncovered a set of generic organizing principles: Most of these systems are scale-free while at the same time modular, resulting in a hierarchical architecture. The structure of the protein domain network, where individual domains correspond to nodes and their co-occurrences in a protein are interpreted as links, also falls into this category, suggesting that domains involved in the maintenance of increasingly developed, multicellular organisms accumulate links. Here, we take the next step by studying link based properties of the protein domain co-occurrence networks of the eukaryotes *S. cerevisiae*, *C. elegans*, *D. melanogaster*, *M. musculus *and *H. sapiens*.

**Results:**

We construct the protein domain co-occurrence networks from the PFAM database and analyze them by applying a *k*-core decomposition method that isolates the globally central (highly connected domains in the central cores) from the locally central (highly connected domains in the peripheral cores) protein domains through an iterative peeling process. Furthermore, we compare the subnetworks thus obtained to the physical domain interaction network of *S. cerevisiae*. We find that the innermost cores of the domain co-occurrence networks gradually grow with increasing degree of evolutionary development in going from single cellular to multicellular eukaryotes. The comparison of the cores across all the organisms under consideration uncovers patterns of domain combinations that are predominately involved in protein functions such as cell-cell contacts and signal transduction. Analyzing a weighted interaction network of PFAM domains of Yeast, we find that domains having only a few partners frequently interact with these, while the converse is true for domains with a multitude of partners. Combining domain co-occurrence and interaction information, we observe that the co-occurrence of domains in the innermost cores (globally central domains) strongly coincides with physical interaction. The comparison of the multicellular eukaryotic domain co-occurrence networks with the single celled of *S. cerevisiae *(the overlap network) uncovers small, connected network patterns.

**Conclusion:**

We hypothesize that these patterns, consisting of the domains and links preserved through evolution, may constitute nucleation kernels for the evolutionary increase in proteome complexity. Combining co-occurrence and physical interaction data we argue that the driving force behind domain fusions is a collective effect caused by the number of interactions and not the individual interaction frequency.

## Background

Many complex systems can best be analyzed as networks where the basic building blocks of the system are represented as nodes and their interactions as links: Recent studies of systems as disparate as the network of scientific co-authorships, sexual contacts and the World-Wide-Web have revealed unexpected similarities, suggesting that their structure and growth is ruled by a set of generic organizing principles [1,2]. A variety of biological systems, like food webs and the various biochemical interactions between genes, proteins and metabolites, have been found to exhibit similar large-scale traits [3-6]. The most prominent is the scale-free property of the connectivity distribution. When combined with a modular structure, the resulting network consists of a hierarchy of interwoven clusters [7-10].

Protein crystallography reveals that the fundamental unit of protein structure is the domain. Independent of neighboring sequences, this region of a polypeptide chain folds into a distinct structure and mediates biological functionality [11]. Comparing domain architectures of proteins in multicellular organisms evidence emerged that preexisting domain architectures have predominantly been supplemented with single domains at their terminal sites [12,13].

Functional links between proteins have also been detected by analyzing the fusion patterns of protein domains. Two separate proteins A and B in one organism may be expressed as a fusion protein in other species. A protein sequence containing both A and B is termed a Rosetta Stone sequence. However, this framework only applies in a minority of cases [14].

The structure of the protein domain network, where individual domains are nodes and their co-occurrences in a protein are interpreted as links, also displays a scale-free structure [15-17]. Domains that are involved in cell-cell interactions, signal transduction and cell differentiation have been found to accumulate links, reflecting increasing complexity of the organisms specific evolutionary development in going from bacteria to eukaryotes.

In a recent study [18], we classified yeast proteins as being either *globally *or *locally *central according to the number and density of links in their network neighborhoods. In particular, we applied an iterative decomposition method (see Methods) that systematically uncovered core networks with nodes having degrees of at least *k*. In nesting through the different cores, we gradually defined highly connected proteins in the innermost cores as *globally *central while we call proteins that have been placed in cores on the periphery *locally *central. This categorization allowed us to demonstrate that *globally *central proteins participate in a substantial number of complexes while simultaneously displaying a high level of evolutionary conservation. Here, we apply this core decomposition method to study the properties of the protein domain co-occurrence networks of the eukaryotes *S. cerevisiae*, *C. elegans*, *D. melanogaster*, *M. musculus *and *H. sapiens*, allowing us to classify the various domains as either globally or locally central. In going from the single celled Yeast to the considered highly evolved multi cellular organisms we find that the number of globally central domains increases with the organisms level of evolutionary development. Also the overlap network which consists only of the nodes and links shared by *all *the organisms specific cores reveals those domain fusions that have been preserved through evolution. Comparing the co-occurrence networks to the physical protein domain interaction network of *S. cerevisiae *[17,19] we find that links that appear in the innermost cores of the co-occurrence network of higher eukaryotes strongly coincide with physical interactions. The co-occurrences of domains that make them end up in the innermost cores of the co-occurrence networks might represent evolutionary patterns that serve as a putative proteome backbone. Since we find the driving force behind fusion events not to be a high frequency of interactions between a given protein domain pair but a large number of individual interaction partners, we conclude that links appearing in the innermost cores of the co-occurring networks are the result of underlying important domain interactions.

## Results

### Statistics of domain networks

**Figure 1:**
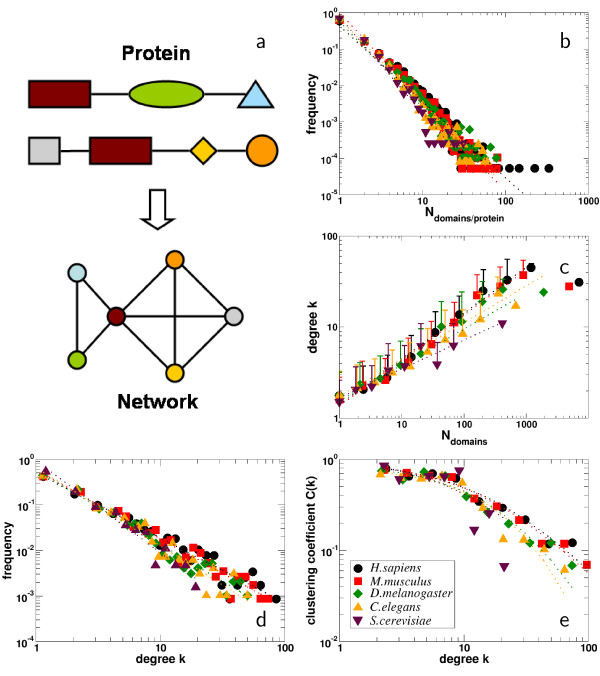
**Basic statistics of domain occurrence networks**. **(a) **All domains co-occurring in a single protein are represented as a fully connected unweighted clique in the network. **(b) **Determining the number of domains each protein contains in *H. sapiens*, *M. musculus*, *D. melanogaster*, *C. elegans*, and *S. cerevisiae*, we observe power-laws *P*(*N*) ~ *N*^-*δ *^in frequency distributions thus obtained (see Table 1 for detailed values). This inhomogeneity in domain architectures suggests that the vast majority of proteins in all organisms considered contains only one domain. **(c) **Counting the occurrence of each domain in the proteomes of the organisms under consideration, we find a positive power-law dependence from the mean number of co-occurring domains – the degree – ⟨*k*⟩ ~ *N*^*ε*^, suggesting that on average frequent occurrence of a domain coincide with the participation in various domain architectures (see Table 1 for detailed values). **(d)**. The domain networks of *H. sapiens*, *M. musculus*, *D. melanogaster*, *C. elegans*, and *S. cerevisiae *display scale-free behavior, a network feature which is characterized by the power-law in the degree distribution *P*(*k*) ~ *k*^-*θ *^[15] (see Table 1 for detailed values). **(e) **The network's inherent modularity is indicated by the presence of a power-law dependence between the clustering coefficient and the degree as a generalized Zipf-law ⟨*C*(*k*)⟩ = *α*(*β *+ *k*)^-*γ *^(see Table 1 for detailed values). With respect to (b,c,d,e), we observe that the organisms specific distributions differ by their individual power-law exponents, indicating their levels of evolutionary development.

Table 1 summarizes basic statistics of the domain co-occurrence networks of *H. sapiens*, *M. musculus*, *C. elegans*, *D. melanogaster *and *S. cerevisiae*. All the domain co-occurrence networks have a major component containing the vast majority of the nodes, co-existing with many small, connected components. Both the average degree ⟨*k*⟩ and the clustering coefficients ⟨*C*⟩ of the networks gradually increase with elevated level of the organisms development. Determining the number of domains *N *proteins in the proteomes of *H. sapiens*, *M. musculus*, *C. elegans*, *D. melanogaster *and *S. cerevisiae *contain, we observe the presence of power-laws in frequency distributions thus obtained, *P*(*N*) ~ *N*^-*δ *^(Fig. 1b). This result confirms that the vast majority of proteins contains only a single domain [20], while a minority accumulates more domains. In Fig. 1c, we find that *N *shows a positive power-law correlation from the mean number of co-occurring domains – the degree – ⟨*k*⟩ ~ *N*^*ε*^, suggesting that on average frequently occurring domains are combined with an increasing number of changing partners. All co-occurrence networks display a scale-free degree distribution [15] (Fig. 1d), as exemplified by the presence of power-laws, *P*(*k*) ~ *k*^-*θ*^. Similarly, we find a power-law dependence of the clustering coefficient from the degree exemplified by a generalized Zipf-law ⟨*C*(*k*)⟩ = *α*(*β *+ *k*)^-*γ*^, indicating the network's inherent modularity (Fig. 1e). As summarized in Table 1, the dependence of the power-law exponents in Figs. 1b–e well mirrors the level of an organisms evolutionary development. In particular, the higher the level of the organisms development, the smaller the values we find for *δ *(Fig. 1b), *θ *(Fig. 1d) and *γ *(Fig. 1e). In turn, we find the opposite for *ε *(Fig. 1c). Obviously, the increase in complexity of the organisms development coincides with an elevated networks heterogeneity. Since we find similar numbers of domains in the higher eukaryotes (Table 1), we assume that an elevated degree of development presumably is increasingly obtained by frequent domain combinations.

**Table 1:** Statistics of domain networks The domain networks of *S. cerevisiae*, *C. elegans*, *D. melanogaster*, *M. musculus *and *H. sapiens *all contain a major component (*mc*) which incorporates the majority of the domains (), coexisting with many small, connected components (*N*_*cc*_). The number of edges () of the main component, the mean node degree ⟨*k*⟩ and the mean clustering coefficient ⟨*C*⟩ gradually increase with the organisms level of development. Referring to Fig. 1, the frequency distributions of the number *N *of domains per protein (Fig. 1b) follows a power-law *P*(*N*) ~ *N*^-*δ*^. Similarly, we find a power-law in the distributions of the occurrence of domains *N *and their mean degree ⟨*k*⟩ in the organism specific co-occurrence networks *k *~ *N*^*ε *^(Fig. 1c). The degree distributions of the organisms specific co-occurrence networks can be approximated by a power-law *P*(*k*) ~ *k*^-*θ *^(Fig. 1d). Similarly, we approximated the degree dependence of the clustering coefficient by a generalized Zipf-law ⟨*C*(*k*)⟩ = *α*(*β *+ *k*)^-*γ *^(Fig. 1e).

organism	*N*_*cc*_			⟨*k*⟩	⟨*C*⟩	*δ*	*ε*	*θ*	*α*	*β*	*γ*
*H. sapiens*	172	733	4,048	5.52	0.42	2.1	0.5	1.4	61.6	17.9	1.5
*M. musculus*	173	668	3,566	5.34	0.43	2.5	0.5	1.5	67.5	17.1	1.5
*D. melanogaster*	172	506	2,274	4.49	0.39	2.3	0.4	1.6	1,407.8	34.7	2.7
*C. elegans*	167	495	2,120	4.28	0.34	2.4	0.4	1.7	3,886.7	30.9	2.5
*S. cerevisiae*	177	175	516	2.95	0.32	2.6	0.3	2.0	39.4	11.3	1.5

### Cores of domain networks

**Figure 2:**
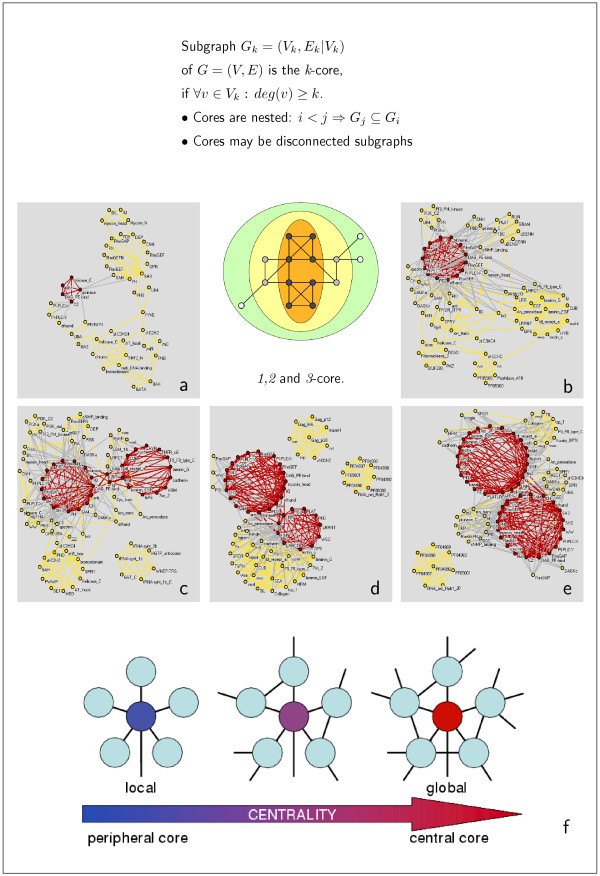
**Cores of the domain co-occurrence networks**. The *k*-core of a graph is defined as the largest subgraph where every node has at least *k *links. For each choice of *k*, we determine the *k*-cores by iteratively pruning all nodes with degree lower than *k *and their incident links. In the schematic representation, the 1-core consists of all the nodes while the 3-core only contains the nodes on orange background. Panels a-e show the 2 innermost *k*-cores (red: 1-core and yellow: 2-core) of the domain networks mapped for the proteomes of **(a) ***S. cerevisiae*, **(b) ***C. elegans*, **(c) ***D. melanogaster*, **(d) ***M. musculus *and **(e) ***H. sapiens*. **(f) ***Local *vs. *global *centrality. Interpreted as its importance a node is related to its degree and network neighborhood. A hub that is only a member of the outer *k*-cores is defined as locally central (top-left), while nodes (not necessarily the biggest hubs) being-members of the innermost cores are globally central (top-right).

Due to the size of the domain co-occurrence networks considered we find different numbers of *k*-cores. While the networks of *H. sapiens *and *M. musculus *are decomposed into 8 nested *k*-cores, where *k *= 1, ..., 8 we find 6 *k*-cores in *D. melanogaster *and *C. elegans *(*k *= 1, ..., 6). There are only 4 in *S. cerevisiae *(*k *= 1, ..., 4). The placement of a node in a certain core allows an assessment of its meaning for the topology. A hub – a highly connected node – that is only a member of the peripheral *k*-cores is defined as locally central, while nodes (not necessarily the biggest hubs of the whole network) being members of the innermost cores are globally central (Fig. 2f). In Table 2, we compiled lists of the highest connected domains in each core layer, being the set of nodes two consecutive cores do not have in common. Notably, the innermost *k-core *is not populated by the largest hubs, indicating that a high degree alone does not necessarily imply a central placement in the network. In fact, we observe that with even a considerably low degree domains can be placed in the innermost cores.

**Table 2:** Five most connected domains in the innermost core-layers using their degree *k *in the full domain networks of *S. cerevisiae*, *C. elegans*, *D. melanogaster*, *M. musculus *and *H. sapiens*. Notably, the innermost layer *l *(equivalent to the innermost core) is not populated by the largest hubs, indicating that a high degree alone does not necessarily imply a central placement in the network. In fact, we observe that with even a considerably low degree *k *domains are placed in the inner cores, a domain specific feature we call *globally *central. We also observe highly connected domains that only make it to the outer cores, indicating a *locally *central position in the respective networks.

*S. cerevisiae*			*C. elegans*			*D. melanogaster*			*M. musculus*			*H. sapiens*		
domain	*l*	*k*	domain	*l*	*k*	domain	*l*	*k*	domain	*l*	*k*	domain	*l*	*k*

SH3	4	16	pkinase	6	44	pkinase	6	50	pkinase	8	64	pkinase	8	74
pkinase	4	13	PH	6	38	SH3	6	41	EGF	8	60	EGF	8	64
C2	4	11	SH3	6	33	PH	6	40	PH	8	49	PH	8	59
DAG PE-bind	4	4	efhand	6	27	ank	6	38	SH3	8	49	SH3	8	52
pkinase C	4	4	PDZ	6	26	PDZ	6	35	ig	8	44	ig	8	52

PH	3	14	EGF	5	38	zf-C2H2	5	24	fn31	7	31	zf-C3HC4	7	51
helicase C	3	13	ank	5	36	zf-C3HC4	5	23	Idl-recept a	7	26	WD-40	7	39
zf-C3H4	3	10	zf-C3HC4	5	24	UBA	5	23	tsp 1	7	25	tsp 1	7	27
UBA	3	10	ig	5	24	helicase C	5	22	zf-CCHC	7	24	UBA	7	26
myb DNA-bind	3	10	fn3	5	20	efhand	5	21	WW	7	21	vwc	7	21

WD40	2	11	F-box	4	19	WD-40	4	18	zf-C3HC4	6	48	zf-C2H2	6	31
AAA	2	10	zf-C2H2	4	18	rrm	4	13	WD40	6	31	PHD	6	27
ank	2	8	WD-40	4	17	TPR	4	11	zf-C2H2	6	28	helicase C	6	24
UCH-2	2	6	rrm	4	15	tsp 1	4	10	helicase C	6	22	rrm	6	23
HATPase c	2	6	PHD	4	15	zf-CCHC	4	10	TPR	6	19	TPR	6	23

clathrin	1	3	UBA	3	10	ubiquitin	3	10	PHD	5	25	BRCT	5	18
ENTH	1	2	homeobox	3	10	AAA	3	9	bromodomain	5	19	PWWP	5	14
zf-CCCH	1	2	Kunitz BPTI	3	10	heme 1	3	7	BRCT	5	17	DEAD	5	14
exo-endo-phos	1	2	metallophos	3	9	GTP-EFTU	3	7	SET	5	13	zf-RanBP	5	13
SAP	1	2	Dna J	3	7	kinesin	3	6	DEAD	5	13	myb-DNA bind	5	13

In Fig. 2a–e we show the two innermost cores of the protein domain networks of *S. cerevisiae*, *C. elegans*, *D. melanogaster*, *M. musculus *and *H. sapiens*, respectively. We observe that the increase in the organisms complexity is not only reflected by higher numbers of cores, but the innermost cores differs in size. We find, that *H. sapiens *has 43 domains in the innermost core, while we find 30 in *M. musculus *and *D. melanogaster*, 14 in *C. elegans *and 5 in *S. cerevisiae*. Reflecting the increasing evolutionary development of the underlying organism, the cores are enriched with domains predominantly associated with functions such as cell-cell contact and signal transduction. This observation agrees well with the known evolutionary development from the single cellular *S. cerevisiae *to the multicellular higher eukaryotes. In particular, the innermost core of *S. cerevisiae *(panel a) consists of the following clique of interconnected domains: (i) pkinase and (ii) pkinaseC which are signal transduction domains, (iii) DAG-PE-bind, a domain which binds Diacyl-glycerol, activating the family of the previously mentioned kinases, (iv) HR1 which is involved in binding the small G-protein rho, and (v) C2, a Ca^2+^-dependent membrane-targeting module found in many cellular proteins that are involved in both signal transduction and membrane trafficking.

Nesting through the innermost cores of the more evolved organisms, we find that the initial small innermost core of Yeast is enriched with clusters of densely connected domains (Fig. 2b–e). Obviously, this expansion of the innermost core is mostly caused by domains which are involved in signal transduction and cell-cell contacts as well as cell development, suggesting that the demand to maintain a multicellular organism is the driving force for fusing protein domains. In particular, we observe that domains providing these functions, such as PDZ, efhand, SH2 and SH3 to name a few (Fig. 2b–e, Table 2), increasingly populate the innermost cores. However, the affiliation of one domain to a certain core is not inevitably constant. In fact, we observe that pkinaseC which appears in the innermost core of the Yeast domain network occurs in the 2-core of the network of the multicellular organisms. The affiliation of HR1 to the innermost core of Yeast appears to be the effect of a single protein architecture which contains numerous domains, since this particular domain does not appear in the inner cores of the other organisms. Since the majority of Yeast proteins only has one to two domains, being teamed up with a reasonably large number of domains in a given protein is beneficial for a domain to make it to the innermost cores. In multicellular organisms, the number of proteins with a large number of domains increases, inevitably resulting in an inflation of the inner cores. Consequently, the evolutionary significance of a domain is well reflected in its ability to remain present in the inner cores of different organisms.

### Domain interaction network

**Figure 3:**
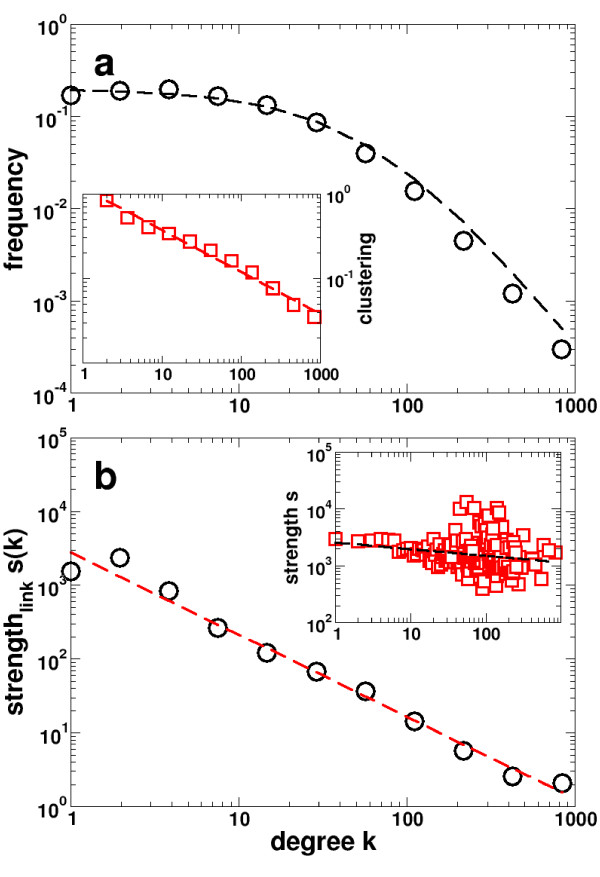
**Statistics of the domain interaction network of Yeast**. The domain interaction network has an average node connectivity of ⟨*k*⟩ = 16.9 along with a reasonably high degree of clustering ⟨*C*⟩ = 0.34. **(a) **The degree distribution of the domain interaction network displays a power-law, following the generalized Zipf-law *P*(*k*) = *α*(*β *+ *k*)^-*γ *^where *α *= 3,406.4, *β *= 67.4 and *γ *= 2.3. The network's inherent modularity is suggested by the presence of a power-law dependence in the average clustering coefficient ⟨*C*⟩ ~ *k*^-*β *^(inset), where *β *= 0.5. **(b) **The average strength *s*_*i *_of each interaction domain *i *displays a power-law (*s*_*i*_(*k*_*i*_) ~ ) over four decades. Obviously, this is an effect of a domains level of interaction, since we only recover a weak decrease of the strength *s*_*i *_toward higher degree *k*_*i*_, *s*_*i *_~ *k*^-0.1^.

Information about protein domain interactions as of the InterDom database [21] constitute an undirected network of Yeast protein domain interactions. In contrast to domain co-occurrence networks, each link has a weight which reflects the frequency of the corresponding interactions relative to a random background distribution [21]. The degree distribution of the domain interaction network (Fig. 3a) is well fitted by a generalized Zipf-law *P*(*k*) ~ *α*(*β *+ *k*)^-*γ*^, suggesting that a few ubiquitous domains (hubs) dominate the web of domain interactions. The network's inherent modularity is expressed by the power-law form of the degree dependent clustering coefficient ⟨*C*(*k*)⟩ ~ *k*^-*δ *^(Fig. 3a, inset). In order to combine the impact of topology and weights, Barrat et al. [22] introduced a series of measures that allowed a more significant assessment of the impact weights have on the networks statistical properties. In a weighted representation of a domain interaction network the strength of a domain is the sum of the weights *w*_*ij *_carried on each link,  (Note, that the strength *s*_*i *_of a node *i *is the degree *k*_*i *_if we consider a network where all weights are 1). In the inset of Fig. 3b, we observe that the strength of the average interaction weakly decreases with increasing degree *k*. Assessing the distribution of weights, we define the average strength per link by . This measure allows us to observe a decreasing trend of *s*(*k*) with *k *(Fig. 3b) as a power-law, suggesting that domains with many interaction partners only occasionally interact with each partner.

**Figure 4:**
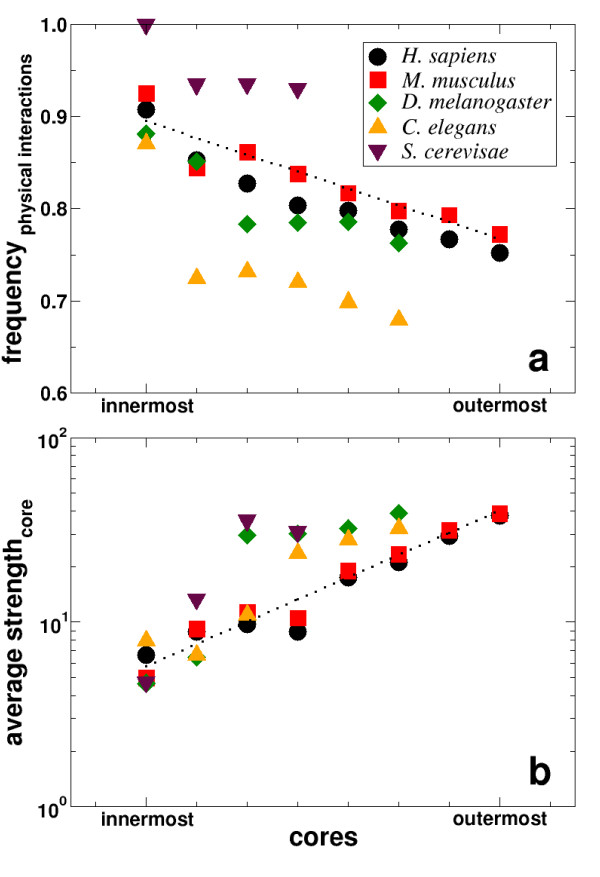
**Driving force behind fusion proteins**. **(a) **Nesting toward the innermost core for the eukaryotes *S. cerevisiae*, *C. elegans*, *D. melanogaster*, *M. musculus *and *H. sapiens *we find that the co-occurrence links increasingly coincide with links in the Yeast protein domain interaction network. **(b) **The interaction strength *s *of domains is the sum of the interaction weights of all links a domain is involved in the corresponding cores of the respective co-occurrence networks. Averaging over the size of the corresponding cores, the average interaction strength decreases toward the innermost cores.

How is then the domain interaction network related to the domain co-occurrence network? In each core of the domain co-occurrence networks, we calculated the fraction of links present in the Yeast domain interaction network. Fig. 4a shows these frequencies of links for the eukaryotes *S. cerevisiae*, *C. elegans*, *D. melangaster*, *M. musculus *and *H. sapiens*, all displaying a decreasing trend that a fusion is accompanied by the physical interactions of domains when going from the innermost to the outermost core. Calculating the mean strength of domains based on the links that are present in the different cores by superimposing the respective weights *w*_*ij *_from the domain interaction network, we observe an ascending trend when nesting outwards toward the periphery of the domain co-occurrence networks (Fig. 4b). We interpret the observations that (i) domains which appear in the inner cores likely physically interact with (ii) a low average strength as follows: Domains with numerous interaction partners have an elevated chance of being fused in a higher eukaryote, while domains which interact frequently are less likely to be fused. Indeed, the innermost cores display the lowest average strengths, confirming that the driving force behind the fusion of a domain pair is not their frequent interaction, but rather the engagement of the two domains in a multitude of interactions with other domains.

### Overlap of domain network cores

**Figure 5:**
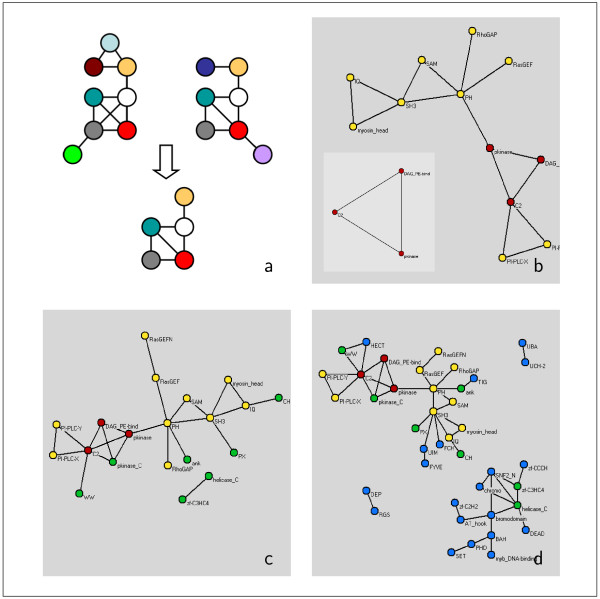
**Overlap of domain co-occurrence networks**. **(a) **We define the overlap of two networks as the edges, and their concomitant nodes, common to both networks. **(b) **The overlap of the four innermost *k*-cores of the co-occurrence domain graphs of *S. cerevisiae*, *C. elegans*, *D. melanogaster*, *M. musculus *and *H. sapiens *only shows a small number of conserved edges (red: 1-core, yellow: 2-core, green: 3-core, blue: 4-core). The overlap of the 1-cores consists of a fully connected kernel populated by signaling domains. Nesting outward in the overlap of the 2, 3, 4-cores (**(b),(c)**), domains that are responsible for signal transduction such as zinc-fingers and cell-cell contacts are dominating.

Many of the domains appear ubiquitous to the innermost eukaryotic cores of the co-occurrence network (see e.g. Fig. 2). As already mentioned, the evolutionary and functional significance of a domain is indicated by its presence in the innermost cores of many organisms. Similarly, the conservation of links represents an evolutionary and functional signal. So far, it is unclear if *links *between these globally central domains have been preserved in all the eukaryotes. Fig. 5b–d shows the domain links simultaneously present in the four central cores of *S. cerevisiae*, *C. elegans*, *D. melanogaster*, *M. musculus *and *H. sapiens*. Note that all the links in Fig. 5 have been preserved during evolution, suggesting the existence of a deeper reason why these domains seemingly always appear together in proteins. To be more specific: The central core of this domain-overlap network consists of a triangle set up by C2, pkinase and DAG-PE-bind (Fig. 5b, red nodes). Nesting outward through the cores, we find a further accumulation of triangles, all established by such prominent domains as PH, SH3 and RasGEF (Fig. 5b, yellow and red). In the subsequent overlap of cores (Fig 5c and 5d) we observe the presence of the important signaling domains zf-CCCH, zf-C2H2 and zf-C3HC4 from the zinc-finger family. Note that *all *of the (co-occurrence) links in the overlap networks correspond to physical interactions between the domains, *i.e*. the fraction of links in the overlap cores that are present in the interaction network is 1.0.

## Discussion & conclusions

Although the PFAM database provides comprehensive domain information, it covers only a part of the considered proteomes. Similarly, the determination of putative domain interactions depends on the quality and completeness of the underlying sets of protein interactions. Yet, the heterogeneity of scale-free networks indicates that the general characteristics of domain co-occurrence and interaction networks are independent of the webs actual size [17]. In particular, such networks are governed by the presence of highly connected hubs and cohesive areas, factors that not only influence their integrity but also the determination of *k*-cores. Since biological networks have been found to be stable upon random perturbations, we expect that the addition of new data will not dramatically impact our findings. The idea of analyzing the protein domain co-occurrence network as a sequence of nested cores and comparing the overlap between the central cores of eukaryotic organisms with increasing level of evolutionary development, gives new and fundamental insights into the qualitative arrangement and evolutionary utilization of the proteome. The evolutionary trend toward multicellularity requires proteomes capable of new and additional complex cellular processes such as signal transduction or cell-cell contacts. On a node based level, this trend toward higher complexity is reflected by an considerable heightened connectivity of domains that support such functions in multicellular organisms [15]. Turning our attention to a link-based level, panels in Fig. 2 suggest an analogous result. The steadily increasing size of the innermost *k*-cores allows us to observe that the demand of maintaining complex cellular process does not only impact the level of single domains, but also operates on a combinatorial level of domain arrangements. Nevertheless, many protein families involved in inter- and intracellular signaling pathways, apoptosis [23], development, and immune and neural functions [12,13], are indeed augmented in *H. sapiens *relative to *D. melanogaster *and *C. elegans*. Although human phenotypic complexity by far exceeds that of *D. melanogaster *and *C. elegans*, proteome dimensions remain surprisingly similar, allowing us to conclude that increased functional complexity is not simply a matter of proteome size but strongly underlines the role of innovations on the level of domain (re-) arrangements.

In fact, a significant portion of the protein architecture is found to be homolog in *H. sapiens *and *D. melanogaster *while substantial innovation in the creation of new protein architectures also has been detected [12]. The expansion of selected domain families and the accompanying evolution of complex domain architectures by joining presumably pre-existing domains coincides with the increase in the organisms level of evolutionary development. In particular, changes in the domain architectures are the consequence of a cellular mechanism commonly known as 'domain shuffling', appearing in different disguises [20]. In simple cases of creating a new domain architecture, domains are simply inserted in already preexisting domain arrangements, a mechanism known as domain insertion while domain duplication refers to the internal duplication of at least one domain in a gene. Comparing domain architectures of proteins in multicellular organisms evidence emerged that preexisting domain architectures have been supplemented with single domains at their terminal sites, another mechanism that is known as domain accretion [13]. Our results do not favor one mechanism over the other. Yet, the panels in Fig. 2 support the assumption that domain (re-)arrangements massively helped to evolve complex proteomes that are capable to maintain complex cellular processes, that have not been possible with the extension of single protein domain families alone. In the same way, network patterns we obtained from the comparison of cores which appear in all organisms under consideration will not tell us which mechanism predominantly gave rise to their emergence. Yet, we see that such small sized network patterns (see Fig. 5) presumably represent a repository of domain combinations around which the individual proteomes unfolded. These patterns predominately contain domains that play dominant roles in proteins which are essential for the inner workings of a multicellular organism, presumably serving as a possible backbone for the evolution of proteins mainly involved in signal transduction and cell-cell contacts.

The decomposition of the domain co-occurrence networks into *k*-cores allows us to uncover those sets of domains that are embedded in densely connected areas of the networks. The high connectivity as well as the nature of the partners those domains appear with indicate a central topological and functional role in the proteome of the considered organisms. Nesting toward the innermost cores the significance of these links is supported by the observation that pairs of co-occurring domains increasingly are present as physical interactions in Yeast. Utilizing the combined information of the co-occurrence network and the physical interaction network, we also find that domains tend to interact infrequently if they have many different interaction partners. In contrast, we observe that domains interact increasingly frequently once they have a small number of partners. Although we considered domain fusions on an indirect and qualitative basis this series of observations suggests that the driving force behind domain fusion events is not frequent interactions. In fact, it seems that the *number *of interactors, the connectivity, of the domains mainly influences a domains propensity to fuse with other interactors. The trend to spatially organize otherwise randomly diffusing domains might help to organize the flow of information in cells. Concluding, we find that domain fusion is a tool to superannuate the random diffusive interaction of a domain pair by embedding them in an architecture which ensures their interactions that would be difficult by random diffusion in a cell alone.

## Methods

### Network representation

An undirected unweighted network of *n *nodes is conveniently represented as an symmetric *n *× *n *adjacency matrix *A *= (*a*_*ij*_), where *a*_*ij *_= 1 if there exists an edge between nodes *i *and *j *and *a*_*ij *_= 0 otherwise. In a weighted network, the adjacency matrix reads as *A *= (*a*_*ij*_*w*_*ij*_), where *w*_*ij *_represents the weight of edge *ij*. Consistently, the degree being the number of neighbors a node *i *has is . As a generalization of a nodes degree the strength *s*_*i *_of a node i is defined as [22].

### Proteome databases and domain co-occurrence network

The Integr8 database [24,25] provides comprehensive statistical and comparative analyzes of the proteomes of fully sequenced organisms. Every predicted protein is annotated with the domains it contains, utilizing the combined efforts of different domain sequence sources. For our analysis, we focused on the domain data retrieved from the PFAM database, a reliable collection of multiple sequence alignments of protein families and profile hidden Markov models [26]. We construct the protein domain networks by considering all PFAM domains (or nodes) that are co-occurring in a protein to be a fully connected clique of undirected links (see Fig. la). In Integr8 we find 19,061 proteins that have a PFAM annotation in *H.sapiens*, as well as 18, 953 of *M. musculus*, 9, 785 of *D. melanogaster*, 12, 587 of *C. elegans *and 3, 791 of *S. cerevisae*. Although domain combinations *ij *potentially occur repeatedly in a proteome, we assign weight *w*_*ij *_= 1 to every link between domains *i *and *j*. Following this procedure, we generated domain networks for the proteomes of *H. sapiens*, *M. musculus*, *C. elegans*, *D. melanogaster *and *S. cerevisiae*.

### Domain interaction data

The Interdom database [21,27,28] provides computationally derived putative domain interactions of Yeast. Based on PFAM domain information [26] for each set of protein interactions including pairwise protein interactions, protein complexes and Rosetta Stone sequences the presence of potential domain interactions is determined. The occurrence of a domain interaction in each protein interaction set is evaluated by comparing the observed frequencies to a random background model. A score thus obtained reflects the abundance of a particular pair-wise domain interaction, allowing the assessment of the reliability and the significance of the considered domain interaction. Considering these scores as weights *w*_*ij *_of interactions between protein domains *ij*, we generate an undirected network of 3, 353 domains that are embedded in 28, 339 weighted interactions.

### Network degree distribution

The simplest way to characterize a network is by the degree *k *(or connectivity) of the nodes, reflecting the number of neighbors each node has. Accordingly, we define the average degree of a network as ⟨*k*⟩ = (1/*N*) *k*_*i*_, where *N *is the total number of nodes. Recent studies of biological networks have produced compelling evidence that the network degree distribution – the probability that a node has *k *neighbors – is scale-free with the functional form *P*(*k*) ~ *k*^-*γ *^[1,9]. An important feature of the power-law distribution is the presence of a minority of nodes, carrying a vast number of connections, called 'hubs'.

These hubs exhibit an increased propensity to be simultaneously lethal and conserved through evolution [17,29,30], thus playing a crucial role for the integrity of protein interaction networks.

### Network clustering

Another important feature of biological networks is their tendency to exhibit cohesive areas: The clustering coefficient [31] of a node *i *measures the actual number of triangles that node *i *is a member of, relative to the possible number of triangles. Formally, it is defined as



where *n*_*i *_denotes the number of triangles. Accordingly, we define the average clustering coefficient as ⟨*C*⟩ = (1/*N*) *C*_*i*_. The clustering coefficient of a network also carries information about its modular nature, since ⟨*C*⟩ ~ 1 necessitates the presence of tightly interconnected clusters of nodes. Note that the network has a hierarchical architecture when ⟨*C*(*k*)⟩ ~ *k*^-*α*^, allowing the existence of discernible, yet topologically overlapping, functional modules. Apparently, networks with this structure are observed in most types of biological systems where a small subset of hubs play the important role of linking, and hence bridging, the various network modules [9,32].

### *k*-cores

The *k*-core of a graph is defined as the largest subgraph for which every node has at least *k *links (Fig. 2): For each choice of *k*, we determine the *k*-cores by recursively pruning all nodes with degree lower than *k *and their incident links. In particular, we applied the following recursive algorithm: (1) sort nodes according to their present degree, and (2) remove the nodes with degree lower than *k *[18,33,34]. The layer *l*_*k *_of two consecutive cores *k*, *k *+ 1 is defined as the set of nodes that both cores do not have in common, i.e. nodes which only occur in the larger core. Since the innermost core does not have a successive core, we define the innermost core to be equivalent to a layer.
